# LINC00184 involved in the regulatory network of ANGPT2 via ceRNA mediated miR-145 inhibition in gastric cancer

**DOI:** 10.7150/jca.49138

**Published:** 2021-02-22

**Authors:** Hai-yan Piao, Shuai Guo, Haoyi Jin, Yue Wang, Jun Zhang

**Affiliations:** 1 Medical Oncology Department of Gastrointestinal cancer, Liaoning Province Cancer Hospital & Institute (Cancer Hospital of China Medical University), No. 44 Xiaoheyan Road, Dadong District, Shenyang City, Liaoning Province, China 110042.; 2 Gastric Cancer Department, Liaoning Province Cancer Hospital & Institute (Cancer Hospital of China Medical University), No. 44 Xiaoheyan Road, Dadong District, Shenyang City, Liaoning Province, China 110042.; 3 Pancreatic and Thyroid Surgery Department, Sheng Jing Hospital of China Medical University, 36 Sanhao St, Heping District, Shenyang City, Liaoning Province, China 110003.

**Keywords:** Gastric cancer, ceRNA network, TCGA, LINC00184, miR-145, ANGPT2

## Abstract

**Background:** Disrupted gene levels are intimately correlated with the occurrence and prognosis of gastric cancer (GC). As genes do not function in isolation, we set out to investigate the possible relationship among mRNA and non-coding RNAs (ncRNAs).

**Materials and methods:** RNA sequencing from 406 cases of GC was acquired through the TCGA database. R packages were utilized to assess differential RNA expression. The competing endogenous RNA (ceRNA) network was predicted using miRcode, miRDB, mirTarBase, Target Scan and constructed by Cytoscape 3.6.1. GO enrichment analysis, KEGG pathway analysis, GSEA, and WGCNA were applied for pathway analysis. The expression of select candidate molecules was confirmed using western blot and RT-PCR in GC cells and tissues. CCK-8, EdU staining, and Transwell assays were conducted to assess the influence of candidate molecules on proliferation and invasion. The gain and loss-of-function were achieved by co-culture with sh-lncRNA, mimics and sh-mRNA. Luciferase reporters were created using the psiCHECK2 vector, and the relative luciferase activity was calculated.

**Results:** Using data from TCGA, we determined differentially expressed RNAs and created a ceRNA regulatory network. Interestingly, we identified a regulatory complex surrounding ANGPT2. We detected that ANGPT2 was highly expressed in GC, which correlated with a worse prognosis. Our findings indicated that ANGPT2 encourages growth, invasion, and epithelial-mesenchymal transition (EMT) in GC. Importantly, miR-145 inhibits ANGPT2 and abrogates its effects. Furthermore, LINC00184, a ceRNA, blocks miR-145, thereby improving ANGPT2-mediated carcinogenesis.

**Conclusions:** Our findings indicate that the LINC00184/miR-145/ANGPT2 pathway has a crucial function in the development of GC and can act as a possible biomarker and targets for GC therapy.

## Introduction

Gastric cancer (GC) is a digestive system malignancy. Even though advances have been made in GC therapy, it is a leading reason behind cancer-associated mortality 1. The high GC mortality rates are credited to the particular biologic characteristics of GC, which include achieving a time-sensitive diagnosis, a late manifestation of clinical symptoms, and an increased rate of invasiveness and metastases 2. Hence, identifying markers and possible molecular mechanisms in GC is necessary. Greater than 98% of transcriptional yield codes for non-coding RNAs (ncRNAs). Shreds of evidence have validated that dysregulated ncRNAs have an essential function in controlling oncogenes or tumor suppressors 3. At present, it is thought that the complicated regulatory system encompassing mRNA and ncRNAs are a possible reason leading to malignancy of gastric cancer 4.

Nowadays, several ncRNAs, including microRNA (miRNAs) and long ncRNAs (lncRNAs), are the main focus of tumor research and have attracted the attention of numerous researchers. LncRNAs and miRNAs play a vital function in tumor development, invasion and metastasis, malignant proliferation, and chemotherapy resistance 5-7. MiRNAs regulate expression mainly at the post-transcriptional level. On the other hand, LncRNAs have increasingly complex functions. For example, the lncRNA HOTIAR can serve as a “scaffold” to stimulate a physical interaction between Snail and EZH2 8. As “guides”, lncRNA MEG3 involves the formation of Protein-DNA-RNA triplex structures and thus participates in the transcriptional regulation of TGF-β 9. As “decoys”, LncRNAs are bound to miRNAs or protein 10, 11. Noteworthy, one explanation is that lncRNAs are competing endogenous RNAs (ceRNAs), which competitively binds miRNA with mRNA to control mRNA expression 12. Gao S* et al.* reported lncRNA ROR functioned as a ceRNA to regulate Nanog expression by sponging miR-145 in pancreatic cancer 13.

In this study, we acquired data from normal and tumor samples of GC, and calculated differentially expressed RNAs using R packages. Then, we created the ceRNA networks using Perl. We then concentrated on the LINC00184/miR-145/Angiopoietin-2 (ANGPT2) axis and tried to explore its function in the malignancy of GC. We discovered that the LINC00184/miR-145/ANGPT2 can play a role in inducing carcinogenesis by inducing EMT.

## Materials and Methods

### Data analyses

RNA sequencing and matching medical data were obtained from TCGA (https://portal.gdc.cancer.gov/). Raw data was acquired through the RTCGA Toolbox package (R platform). Overall, 406 samples, including 375 GC and 31 normal tissues, were analyzed.

RNA (including LncRNA and mRNA) and miRNA sequences were acquired from Illumina HiSeq_RNASeq and Illumina HiSeq_miRNASeq platforms, respectively. Perl and R were utilized to evaluate findings.

### Identification of differentially expressed RNAs

Package “edgeR” and “limma” helped determine the differentially expressed lncRNAs (DELs), differentially expressed miRNA (DEMis), and differentially expressed mRNAs (DEMs). Genes present across both packages were used for downstream analysis. *P* < 0.05 and |logFC| ≥ 1 were utilized as cutoff. For this analysis, we largely focused on the up-regulated DELs (uDELs), DEMs (uDEMs), and down-regulated DEMis (dDEMis).

### The construction of ceRNA network and survival analysis

Firstly, we utilized miRcode database to pair the corresponding uDELs and dDEMis. Then, the target mRNAs of dDEMis was acquired using miRDB, mirTarBase and Target Scan. Cytoscape 3.6.1 was utilized to create the ceRNA complex. Survival analysis of uDEMs in the network was conducted with the R “Survival” package. *P* < 0.05 represented the cutoff.

### Gene set enrichment analysis (GSEA)

GSEA was utilized to carry out Gene Ontology (GO) and Kyoto Encyclopedia of Genes and Genomes (KEGG) 14. All 24,991 mRNAs in GC tissue and adjacent non-tumor tissue were assessed. The threshold of *P*<0.05 were utilized to predict possible functions and further investigations.

### Constructing Weighted Gene co-expression network

R package “WGCNA” was performed to construct a co-expression gene network 15. The power function, reliant on the soft-threshold β, was utilized to construct a weighted adjacency matrix. Next, we altered adjacency matrix to Topological Overlap Matrix (TOM). Then, we conducted mean linkage hierarchical clustering using per TOM dissimilarity measure. The minimum amount of genes in the group was set as 30 for the dendrogram, 4 for the deep Split and 0.15 for the module dendrogram and merged modules.

### Tissue samples and cell culture

Overall, 47 GC and non-tumorous adjacent tissues were acquired from people undergoing surgery at the Liaoning Province Cancer Hospital and Institute from 2016 and 2018. Individuals were asked to sign an informed consent before operation. The hospital's Ethics Committee approved study.

GES-1 (gastric epithelial cell line) and SGC-7901 and BGC-823 (gastric cancer cell lines) were acquired through China Medical University (Shenyang, China). Cells were maintained in RPMI 1640, 10% FBS, penicillin and streptomycin (Invitrogen, USA) at 37 °C at 5% CO_2_/1% O_2_. Analyses were conducted a minimum of 3 individual times.

### Real-time reverse transcription polymerase chain reaction (RT-PCR)

RNA was obtained using TRIzol as per established protocol. Promega cDNA core kit (Promega, WI, USA) was utilized to create cDNA from 500 ng RNA. SYBR Master Mixture (Takara Bio, Japan) was utilized to conduct RT-PCR (Roche AG, Switzerland). Every sample was assessed in triplicate. U6 served as control. mRNA fold change across different cell lines was evaluated through 2^-△△CT^ normalization. Sequencing is recorded in Supplemental Table 1.

### Western blot analysis

Protein was loaded onto polyacrylamide gel and moved onto PVDF membranes, which were kept in blocking buffer for 1 hour, and then placed with primary antibodies overnight at 4 °C (ANGPT2, 1:200, Abcam, N-cadherin, Vimentin, E-cadherin and β-catenin 1:200, Boster Biological Technology and β-actin, 1:4,000, Santa Cruz). Membranes were then placed in incubation with secondary antibody (1:5,000, Santa Cruz). β-actin served as control. Protein was quantified through the use of ImageJ (NIH, USA). Final data was represented as an average of 3 independent experiments.

### Immunohistochemistry (IHC)

In brief, 5 μm slices were fixed on poly-L-lysine coated slides. ANGPT2 antibody (Abcam, 1:2000) was used to immunostain the sections, and appropriate biotin-coupled secondary antibodies and immunoperoxidase detection (DAB) substrates (BOSTER, China) and Vectastain ABC Elite kit (Linaris, Germany) were utilized for treatment and observation.

The immunohistochemical score was associated with the staining intensity and ratio of positive cells. The final score obtained from two skilled pathologists, independently. Staining intensity ranged from 0 (negative) to 3 (strong).

### Lentivirus vector system, plasmids, and transfection

Lentiviruses with siRNA that target ANGPT2, LINC00184, miR-145 mimics, and inhibitors were acquired through GeneChem (Shanghai, China). Viruses and polybrene (Abbott Laboratories, USA) were utilized for infection. GC cells were maintained in culture for 72h in medium that contained puromycin for cell screening. The sequencing information was listed in Supplement Table 1.

### Cell Counting Kit-8 assay

Cell Counting Kit-8 (CCK-8) was conducted to investigate cell growth capacity as established guidelines (Dojindo Laboratories, Japan). A microplate reader was used to determine optical density using 450 nm wavelength (Bio-Rad, CA, USA).

### EdU staining

5-ethynyl-2'-deoxyuridine (EdU) assays were also used to investigate the cell growth capacity. GC cells were placed on 96-well plates (0.2 million cells/mL per well) and adhered overnight. Cells were then incubated with EdU for 2 hours with 100 μl per well and fixed using 4% paraformaldehyde for 30 min after transfection. *In vitro* imaging dyeing was conducted by the Cell-Light ™ EdU Apollo ® 488 kit (RioBio, China) as per established protocol.

### Cell cycle

Pre-chilled 1 X PBS used to re-suspended cells. 1× Annexin binding bufer diluted cells to 1×105 cells/ml. Cells were precooled with 70% ethanol at 4 °C overnight before assay.

The cells were subsequently incubated with propidium iodide (PI, Sigma, USA) at 37 °C for 1 h. FLow cytometer and BD FACSuite software acquired the proportion of cells in each phase of the cell cycle.

### Transwell invasion assay

Transwell assay was conducted to establish invasive ability. Top wells were coated using gelatin, and GC cells were seeded onto plates. The bottom wells contained 600 μL FBS (Costar, USA). Methanol, H&E were utilized to fix and label cells for 24 h (Sigma-Aldrich, USA). Then, the top wells were taken away, and cells that migrated were calculated and visualized by a microscope at 100× magnification across 5 fields. The mean cell quantity in every field was characterized through migrated cells.

### Scrape assay

The cells were inoculated in culture inserts (Ibidi, Regensburg, Germany) and cultured to the fusion state. The inserts were removed after incubation for 24 h. PBS washed plates. Wounded monolayer images were collected at 0 and 24 h.

### Immunofluorescence

Cells were fixed using 4% paraformaldehyde in PBS for 10 min and permeabilized with 0.1% Triton-X, 1% BSA in PBS for 30 min. Nuclei were labelled using 4',6-diamidino-2-phenylindole (DAPI). Biomarkers were identified using antisense probes that hybridized at 37 °C for about 16h. Visualization was conducted through the use of ECLIPSE Ni microscope (Nikon, Japan).

### Luciferase assay

Luciferase reporters were constructed using psiCHECK2 vector (Promega). Both constructs include the full 3'UTR of ANGPT2. The predicted miR-145 sites and the complete sequence of LINC00184 was inserted into psiCHECK2. The putative miR-145 binding sequences in ANGPT2 or LINC00184 and their mutant of the binding sites were synthesized and cloned to downstream of the luciferase gene in the psiCHECK2 luciferase vector (Promega, USA). As per established guidelines, luciferase reporters were co-transfected using miR-145 mimics and miR-NC into SGC-7901s through Lipofectamine 2000. Dual-Luciferase Reporter Assay System (Promega) and a microplate reader (Tecan) were performed to quantify luciferase activity.

### Statistical analyses

Statistics were carried out using SPSS (IBM, NY, USA). Findings were conveyed as means ± SD. Data comparison was carried out through Student's t-test or Wilcoxon-signed rank test. More than 2 groups were statistically compared through ANOVA. *P* < 0.05 represented statistical significance.

## Results

### Differentially expressed RNAs in GC

**Figure 1:**
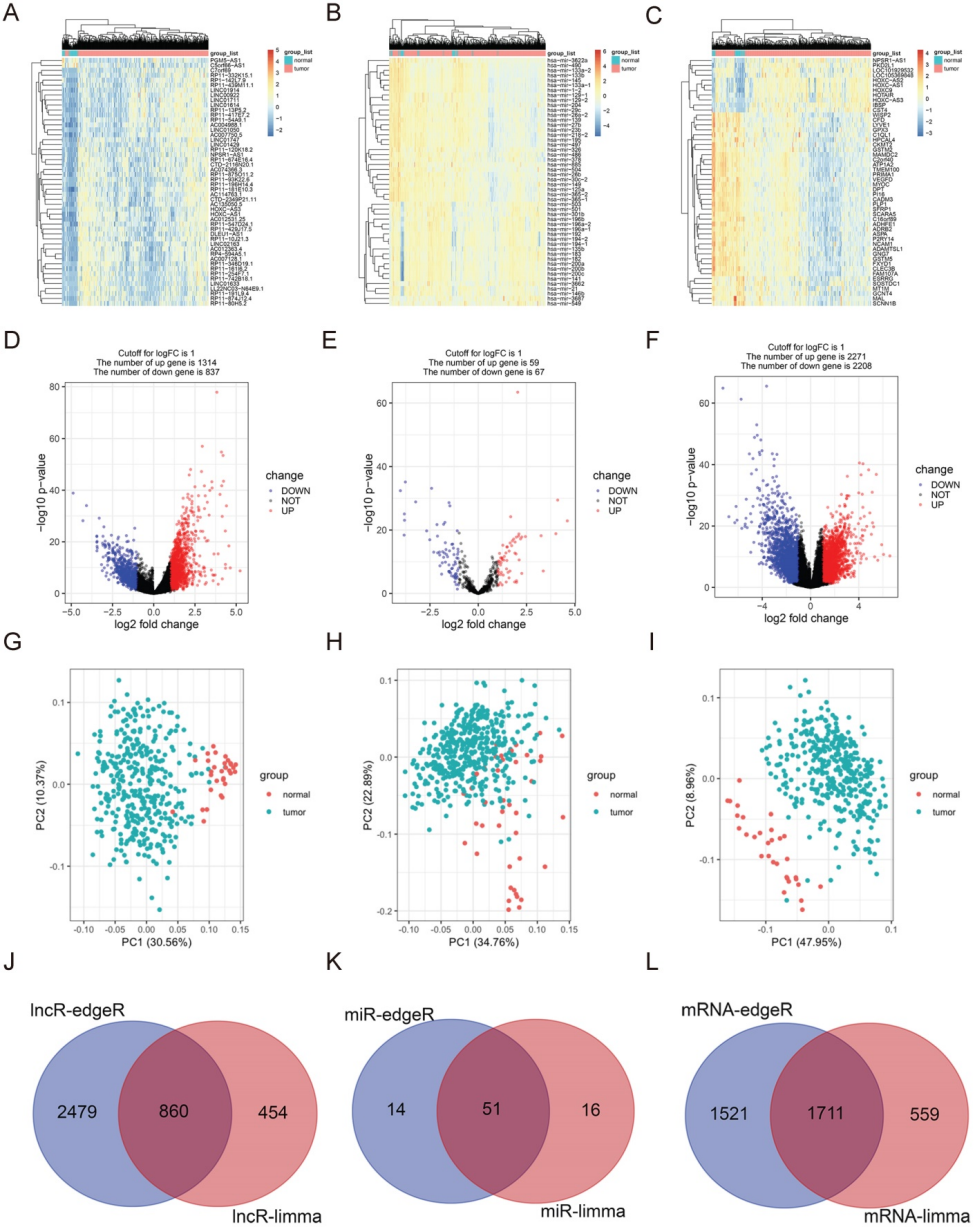
** Differentially expressed RNAs in GC. A-C.** Heatmap of top 50 DELs, DEMis and DEMs, as identified by limma. **D-F.** Volcano plots of differentially expressed DELs, DEMis and DEMs, as identified by limma. **G-I.** PCA of DELs, DEMis and DEMs, as identified by limma. **J.** The up-regulated DELs, determined by limma and edgeR. **K.** The down-regulated DEMis, determined by limma and edgeR. **L.** The up-regulated DEMs, determined by limma and edgeR.

RNA sequencing contains 375 GC and 31 para-carcinoma tissues. EdgeR and limma were used to determine the DELs, DEMis and DEMs (Foldchange ≥ 2, *P* < 0.05). According to limma, 1314 uDELs and 837 dDELs (Fig. 1A, 1D, 1G), 59 uDEMis and 67 dDEMis (Fig. 1B, 1E, 1H), 2271 uDEMs and 2208 dDEMs (Fig. 1C, 1F, 1I) were detected. For edgeR, 3339 uDELs and 925 dDELs (Sup. Fig. 1A, 1D, 1G), 148 uDEMis and 65 dDEMis (Sup. Fig. 1B, 1E, 1H), 3232 uDEMs and 2401 dDEMs (Sup. Fig. 1C, 1F, 1I) were detected. PCA was conducted to assess the relationships (Fig. 1G-I, Sup. 1G-I). Venn diagram demonstrated the intersection of uDELs (Fig. 1J), dDELs (Sup. Fig. 1J), dDEMis (Fig. 1K), uDEMis (Sup. Fig. 1K), and uDEMs (Fig. 1L), dDEMs (Sup. Fig. 1L). In these analyses, we largely concentrated on uDELs, dDEMis and uDEMs. Hence, 860 uDELs, 51 dDEMis and 1711 uDEMs were further explored.

### ceRNA regulatory network

**Figure 2:**
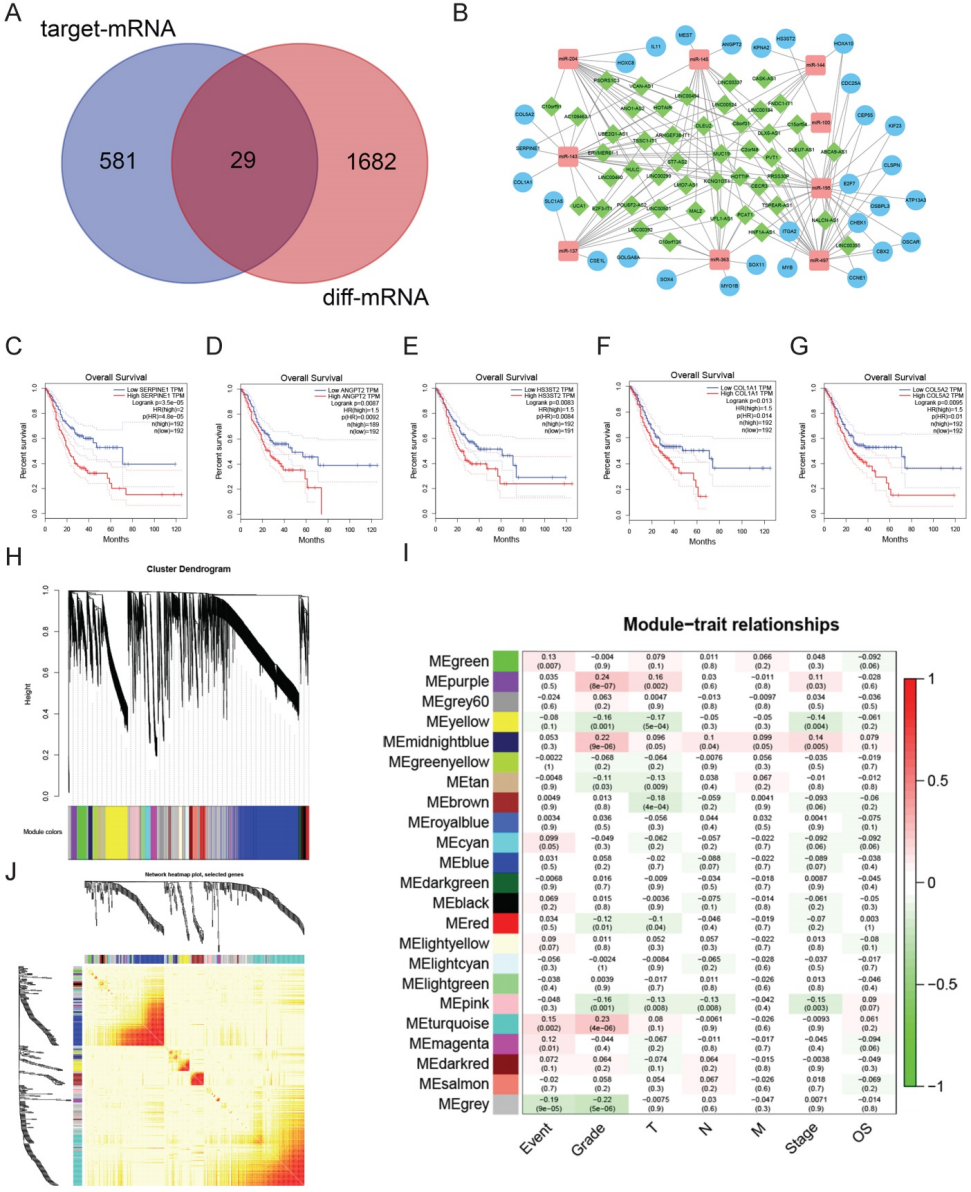
** Constructing ceRNA regulatory network. A.** Venn diagram of uDEMs part of the ceRNA regulation network. Red: All uDEMs in GC. Purple: Target mRNA of dDEMis; Middle: The uDEMs situated in the differential expression and targets. **B.** The ceRNA regulatory network. Green: uDELs; Red: dDEMis; Blue: uDEMs. **C-G.** Five uDEMs are associated with the poor survival of GC. C: SERPINE1; D: ANGPT2; E: HS3ST2; F: COL1A1; G: COL5A2. **H.** Gene clustering tree (dendrogram) acquired by hierarchical clustering of adjacency-based dissimilarity. **I.** Correlation heatmap among modular characteristic gene and clinical features of GC. **J.** TOM plot.

We utilized miRcode to find the relationship between the uDELs and dDEMis. Overall, we found 147 correlations among uDELs and dDEMis, and 48 uDELs that target 13 dDEMis (Table 1). Then, using the miRTarBase, miRDB, and TargetScan database, we performed target prediction for the 13 dDEMis. The findings identified 848 DEMis-DEMs interactions, and 610 target mRNA were obtained. Similar genes between the 610 target mRNA and uDEMs were detected and 29 targets were acquired (Fig. 2A, Table 2). It is worth pointing out that there is no target mRNA of miR-451, miR-187, miR-205, miR-383 in these 29 genes. So, at last, we got 48 uDELs, 12 dDEMis and 29 uDEMs. Cytoscape was utilized to create the ceRNA network to demonstrate the relationship between DELs, DEMis and uDEMs (Fig. 2B).

In order to investigate the clinical significance of the 29 uDEMs mentioned above, R package “survival” was utilized to uncover their clinical significance. The expression of SERPINE1 (*P*=4.8e-05), ANGPT2 (*P*=0.0092), HS3ST2 (*P*=0.0084), COL1A1 (*P*=0.014) and COL5A2 (*P*=0.01) were correlated with worse overall survival (OS) of GC (Fig. 2C-2G).

### Pathway enrichment analysis

We conducted pathway enrichment analysis on all 24,991 mRNAs to elucidate the possible role of identified genes. The sequencing data was converted into an RNK file and uploaded to GSEA. GO analysis reveals the top 5 biological processes (BP) were DNA replication, chromosome segregation, DNA dependent DNA replication, sister chromatid segregation and DNA recombination (Sup. Fig. 2A-E). The top 5 molecular functions (MF) were DNA-dependent ATPase function, DNA helicase function, helicase function, purine NTP-dependent helicase function and microtubule motor function (Sup. Fig. 2F-J). The top 5 cellular components (CC) were condensed chromosome, chromosomal region, condensed chromosome centromeric region, chromosome centromeric region and kinetochore (Sup. Fig. 2K-O).

Overall, 20 KEGG pathways were identified by GSEA, and the top 10 pathways included cell cycle, DNA replication, homologous recombination, mismatch repair, nucleotide excision repair, base excision repair, RNA degradation, one carbon pool by folate, ECM receptor interaction were showed in Sup. Fig. 3.

### Weighted gene co-expression network analysis

In order to explain the genetic functional components and hub genes in GC, the R package “WGCNA” was utilized to create the co-expression network, which included the value of all 24,991 mRNAs. The scale-free network was created using the soft-thresholding power as β = 3 (Sup. Fig. 4A). The network consisted of 23 different co-expression modules, each containing 41 to 1038 genes (Fig. 2H).

The module indicating the highest correlation with GC patients' clinical characteristics was purple, as well as the module with the highest correlation with tumor grading (r = 0.24, *P* = 8 × 10 ^-7^, Fig. 2I). The correlation between modules was created by the TOM plot function (Fig. 2J). The correlation of modules was quantified with characteristic genes as representative. The dendrogram was used to cluster modules and the heatmap revealed the characteristic genetic adjacency of the module (Sup. Fig. 4B).

Module membership (MM) was used to evaluate the correlation between genes and modules as a hub gene, the MM value of ANGPT2 to module purple was 0.876, *P*= 0.008 (SERPINE1,* P*= 0.154; HS3ST2,* P*= 0.336; COL1A1,* P*= 0.208; COL5A2,* P*= 0.269). Besides, Gene Significance (GS) was an important indicator used to evaluate genes and biological characteristics. The GS value of ANGPT2 to tumor grade was 0.909, *P*= 0.006 (SERPINE1,* P*= 0.025; HS3ST2,* P*= 0.103; COL1A1,* P*= 0.040; COL5A2,* P*= 0.006).

### ANGPT2 was determined to be a target of miR-145

Using earlier analysis, we believed that disrupted levels of ANGPT2 may be associated with the malignancy of GC. As per the ceRNA network, miR-145 is a possible regulatory compound for ANGPT2 (Fig. 2B).

**Figure 3:**
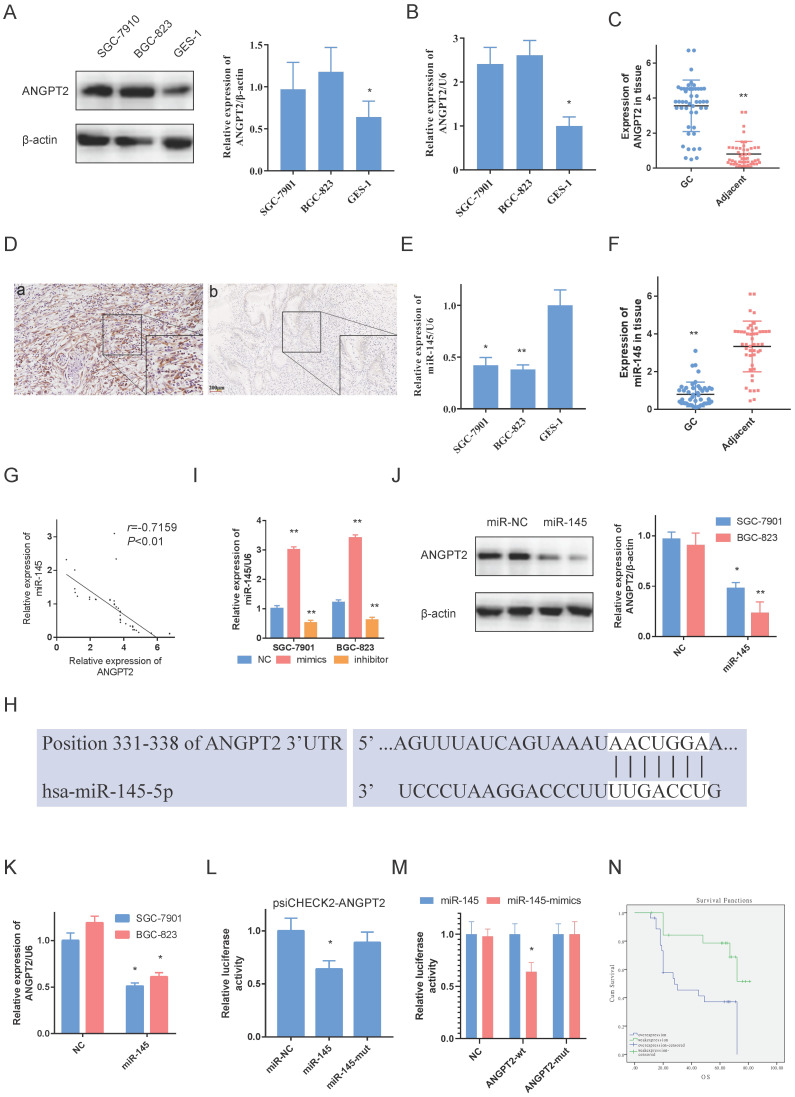
** ANGPT2 is overexpressed in GC and determined as miR-145. A, B.** ANGPT2 was overexpressed in SGC-7901s and BGC-823s compare to it in GES-1. **C.** ANGPT2 was highly expressed in GC. **D.** Representative images of ANGPT2 in GC tissue. (a). ANGPT2 was strongly elevated in GC tissue; but weak-expressed in adjacent non-cancerous samples (b). **E, F.** miR-145 was weak-expressed in GC cells and tissue. **G.** The expressions of ANGPT2 and miR-145 are negatively correlated. **H.** Predicted sites for miR-145 on the ANGPT2 sequence. White nucleotides represent seed sequences of miRNA. **I.** RT-PCR of ANGPT2 in GC cells with mimics and inhibitor of miR-145. **J, K.** GC cells were transfected using miR-145 mimics. Reduced ANGPT2 expression was shown by western-blot and RT-RCR. **L.** Luciferase activities were quantified in SGC-7901s co-transfected using luciferase reporter encompassing ANGPT2 and miR-145 mimics or mutant. Results are represented as ratio to firefly luciferase activity. **M.** miR-145 mimics markedly reduced luciferase activity in ANGPT2-wild not in ANGPT2-mut in SGC-7901 cells. **N.** Kaplan-Meier analysis of ANGPT2 relationship with overall survival in 47 GC patients. Data is exhibited as mean ± SD (n = 3). *P < 0.05, **P < 0.01.

Western blot and RT-PCR results indicated that ANGPT2 was overexpressed in BGC-823s and SGC-7901s in comparison to GES-1s (Fig. 3A, 3B). Besides, we also evaluated ANGPT2 levels in GC and non-tumor adjacent tissue by RT-PCR and IHC. Among the 47 matched specimens, ANGPT2 was seen in all GC tissue and increased compared to adjacent non-tumor tissue (Fig. 3C, 3D).

Further, we identified miR-145 in GC cells and tissue by RT-PCR. As opposed to ANGPT2, miR-145 was highly expressed in GES-1 and adjacent non-cancerous samples (Fig. 3E, 3F). Additionally, it was negatively associated with ANGPT2 in 47 GC tissue samples (Fig. 3G).

The predicted findings from TargetScan indicated that ANGPT2 contains miR-145 targeting sites (Fig. 3H). In order to evaluate the regulatory connection among miR-145 and ANGPT2, we utilized mimics and inhibitor of miR-145 to control miR-145 expression (Fig. 3I). The findings validated that miR-145 negatively regulates ANGPT2 transcriptionally (Fig. 3K) and translationally (Fig. 3J) which were measured by RT-PCR and western-blot, respectively. Additionally, luciferase reporter plasmid psiCHECK2-ANGPT2 was transfected into GCs to assess the influence of miR-145 on ANGPT2. Luciferase activity of psiCHECK2-ANGPT2 was reduced when miR-145 is overexpressed, but not miR-145-mut (Fig. 3L). And the dual-luciferase reporter assay also showed that miR-145 significantly reduced the relative luciferase activity of the wild-type ANGPT2 3'UTR (Fig. 3M). Not surprisingly, the overexpressed ANGPT2 is also associated with worse survival of the 47 GC patients (Fig. 3N). Overall, using NGS and bioinformatics analyses, we treated ANGPT2 as a target. We also validated that miR-145 targeted ANGPT2 by RT-PCR, western blot and luciferase experiments.

### Biology of ANGPT2

**Figure 4:**
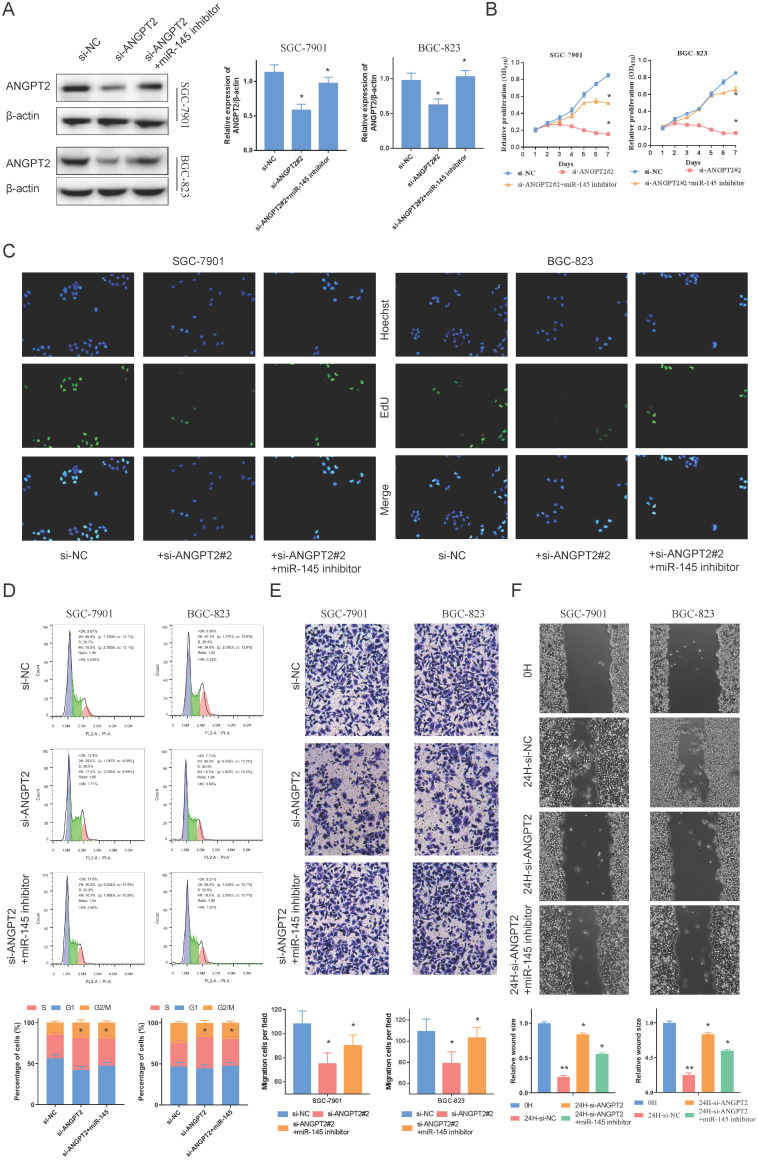
** ANGPT2 reinforces the proliferative and invasion of GC cells. A.** The inhibitor of miR-145 can rescues the inhibitory effect of si-ANGPT2 on ANGPT2. **B.** Cell proliferation assessed in ANGPT2 knockdown and ANGPT2 knockdown + miR-145 inhibitor GC cells by CCK-8. **C.** Cell proliferation assessed in ANGPT2 knockdown and ANGPT2 knockdown + miR-145 inhibitor GC cells by EdU. **D.** The cell cycle progression of GC cells transfected with si-NC, si-ANGPT2, si-ANGPT2 plus miR-145 inhibitor was identifed by flow cytometry assay. **E.** Transwell assays of ANGPT2 knockdown and ANGPT2 knockdown + miR-145 inhibitor. **F.** The scrape assays were used to detect the cell migration ability after transfecting GC cells. Data is exhibited as mean ± SD (n = 3). *P < 0.05, **P < 0.01.

To observe the biology of ANGPT2 and validate the influence of miR-145, we applied RNAi to silence the expression of ANGPT2 in GC cells (Sup. Fig. 5A, 5B). The down-regulation of ANGPT2, stimulated by stable transfection, can be reversed by inhibiting the expression of miR-145 in SCG-7901s and BGC-823s (Fig. 4A). We carried out EdU and Cell Counting Kit-8 (CCK-8) assays to determine the influence of ANGPT2 and miR-145 on cell proliferation. This data indicates that ANGPT2 knockdown could substantially block the growth of GC cells, and influence could be somewhat eliminated by the down-regulation of miR-145 (Fig. 4B, 4C). Furthermore, cell cycle progression was assessed by flow cytometry. Results confirmed that ANGPT2 significantly increased the proportion of SGC-7901 and BGC-823 cells in S phase but decreased the proportion in G2/M phase. However, it could be recovered by miR-145 inhibitor (Fig. 4D). Through Transwell assay, we identified that down-regulating the ANGPT2 can substantially inhibit the invasive ability of SCG-7901s and BGC-823s. When miR-145 was inhibited, the inhibition of invasion was somewhat eradicated (Fig. 4E). Scrape assay showed that the migration distance of cells was shortened with the decrease of ANGPT2, while the migration ability was partially restored after the addition of miR-145 inhibitor (Fig. 4F).

### LINC00184 can crosstalk with miR-145 by direct binding

**Figure 5:**
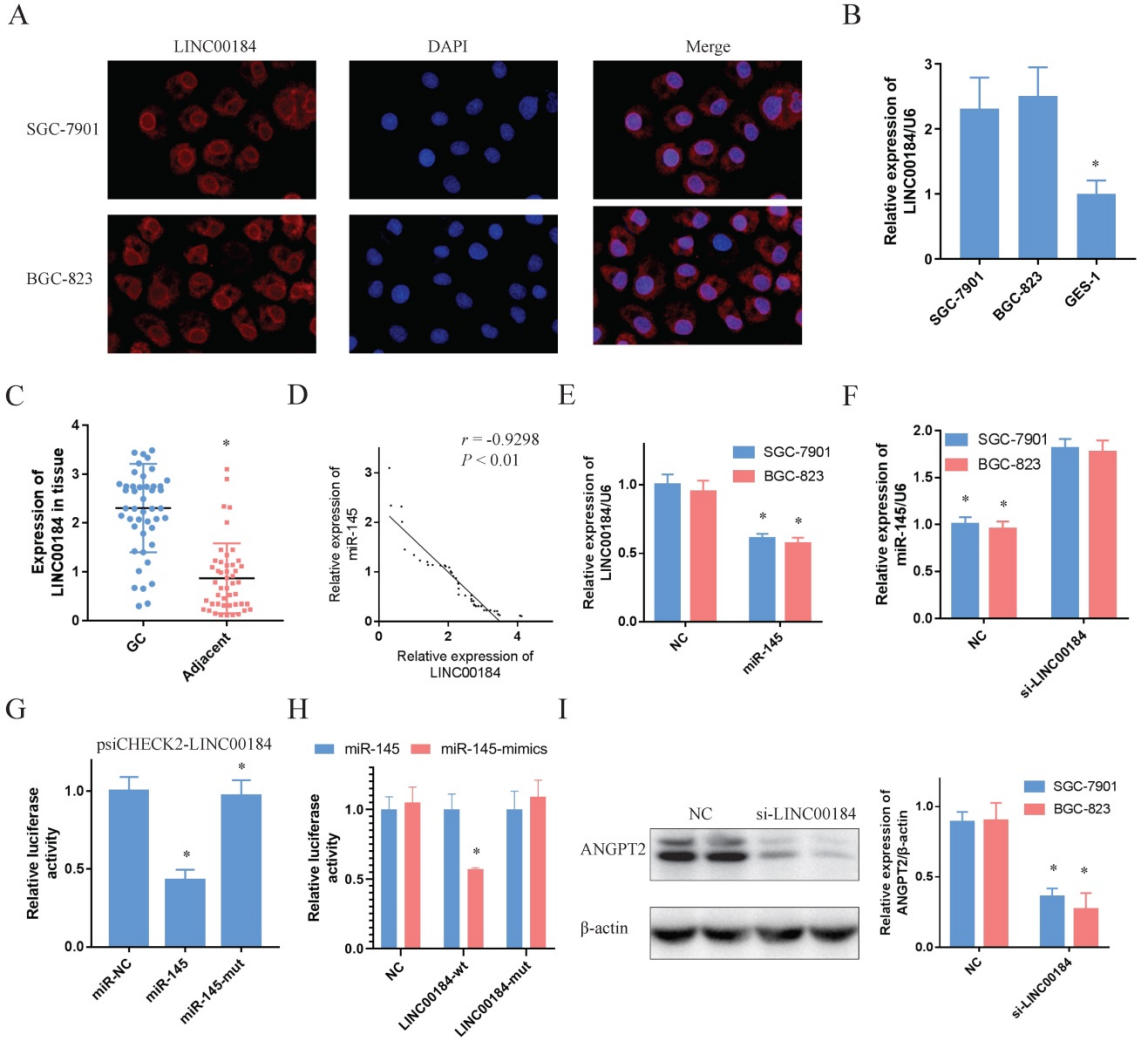
** LINC00184 can crosstalk using miR-145 by direct binding. A.** Immunofluorescence determines that LINC00184 was largely localized in the cytoplasm. **B, C.** LINC00184 was overexpressed in GC cells and tissue. **D.** The expressions of LINC00184 and miR-145 are negatively correlated. **E.** The mimics of miR-145 inhibited LINC00184. **F.** miR-145 expression after knockdown of LINC00184. **G.** Luciferase activity in SGC-7901s co-transfected with luciferase reporter encompassing LINC00184 and miR-145 mimics or mutant. Results represent renilla and firefly luciferase activity ratio. **H.** miR-145 mimics markedly reduced luciferase activity in LINC00184-wild not in LINC00184-mut in SGC-7901 cells. **I.** The expression of ANGPT2 was decreased with the knockdown of LINC00184. Data is exhibited as mean ± SD (n = 3). *P < 0.05, **P < 0.01.

The signal of LINC00184 was primarily localized to the cytoplasm of GC cells, as determined by immunofluorescence and fluorescence microscope (Fig. 5A). Moreover, findings of nuclear/cytoplasmic RNA fractionation by subcellular distribution assay demonstrated the same result (Sup. Fig. 5C, 5D). Similar to ANGPT2, LINC00184 was also overexpressed in GC cells and tissue in comparison to GES-1 and non-tumorous adjacent tissues (Fig. 5B, 5C). Additionally, LINC00184 and miR-145 had a negative relationship in GC tissues (Fig. 5D, *r*=-0.9298, *P*<0.01). In order to determine if LINC00184 may be associated with miR-145 and act as ceRNAs, we evaluated the interaction of their expressions. LINC00184 expression can be down-regulated by the mimics of miR-145 (Fig. 5E). However, miR-145 levels, which were initially low in GC cells, were up-regulated under the intervention of si-LINC00184-1 (Fig. 5F, Sup. Fig. 5E, 5F). Then, we transfected psiCHECK2-LINC00184 into GC cells to identify the possible influence of miRNA on lncRNAs. Overexpression of miR-145, but not miR-145-mut, reduced the luciferase activity (Fig. 5G). And the dual-luciferase reporter assay also showed that miR-145 significantly reduced the relative luciferase activity of the wild-type LINC00184 3'UTR (Fig. 5H). Furthermore, ANGPT2 expression was decreased after knockdown of LINC00184 (Fig. 5I). These findings suggest that LINC00184 directly binds miR-145 and influences its expression.

### LINC00184 act as ceRNAs to regulate cell function

**Figure 6:**
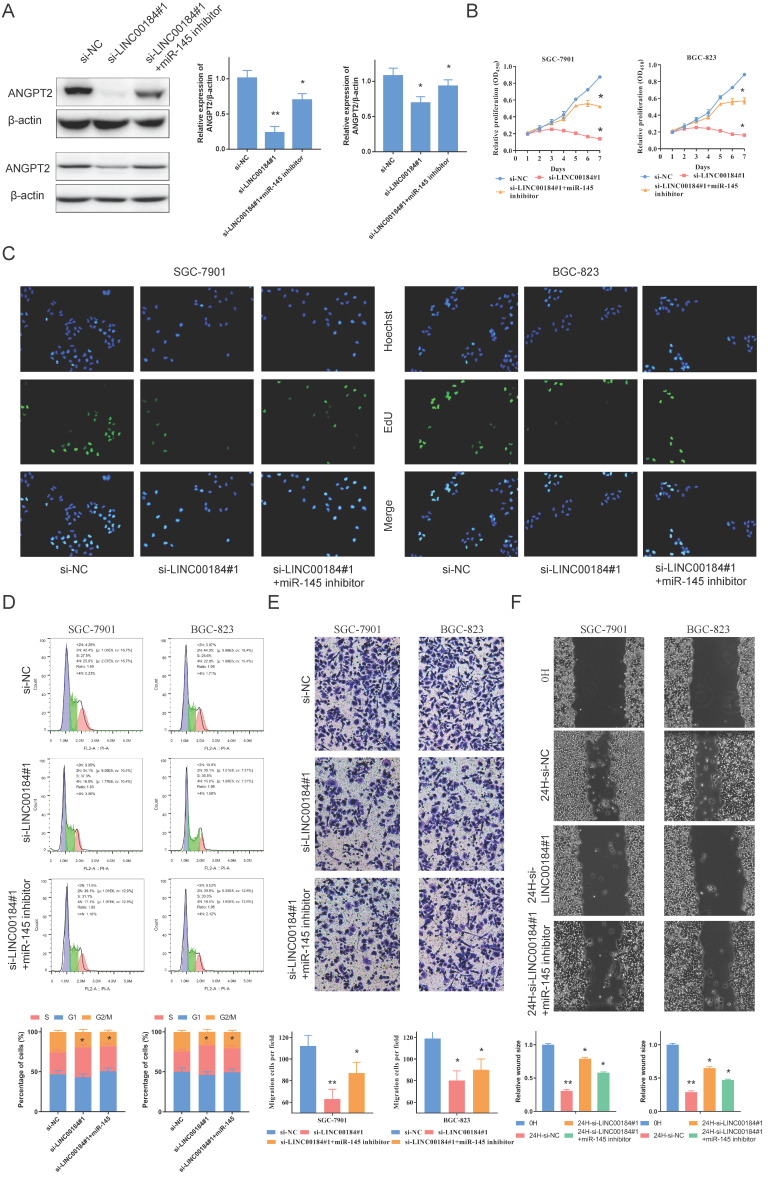
** LINC00184 reinforces the proliferative and invasion of GC cells. A.** The inhibitor of miR-145 can rescue the inhibitory effect of si-LINC00184 on ANGPT2 which were determined western blot. A: SGC-7901; B: BGC-823. **B.** Cell proliferation assessed in LINC00184 knockdown and LINC00184 knockdown + miR-145 inhibitor GC cells by CCK-8. **C.** Cell proliferation assessed in LINC00184 knockdown and LINC00184 knockdown + miR-145 inhibitor GC cells by Edu. **D.** The cell cycle progression of GC cells transfected with si-NC, si-LINC00184, si-LINC00184 plus miR-145 inhibitor was identifed by flow cytometry assay. **E.** Transwell assays of LINC00184 knockdown and LINC00184 knockdown + miR-145 inhibitor. **F.** The scrape assays were used to detect the cell migration ability after transfecting GC cells. Data is exhibited as mean ± SD (n = 3). *P < 0.05, **P < 0.01.

To identify the role of LINC00184, we carried out loss-of -function experiments. A decrease in LINC00184 led to a reduction in the expression of ANGPT2. Additionally, the down-regulation of ANGPT2 induced by si-INC00184 can be reversed by inhibiting the expression of miR-145 in SCG-7901s and BGC-823s (Fig. 6A). CCK-8 and EdU were utilized to assess proliferative capacity. Expectedly, GC's malignant proliferative capacity can be blocked by the low levels of LINC00184, and miR-145 can also somewhat reverse this role (Fig. 6B, 6C). Flow cytometry was employed to assess cell cycle progression. It showed the loss of function of INC00184 could up-regulated the S phase proportion but down-regulated G2/M phase proportion of GC cells. Base on this basis, the addition of miR-145 inhibitor could weaken the inhibition effect of si-INC00184 on the cell cycle (Fig. 6D). LINC00184 down-regulation led to reduced invasive and migration capacity, as measured by Transwell and scrape assay. Not surprisingly, blocking miR-145 abolished this influence (Fig. 6F, 6G).

### LINC00184/miR-145/ANGPT2 axis regulates epithelial-mesenchymal transition (EMT) in GC

**Figure 7:**
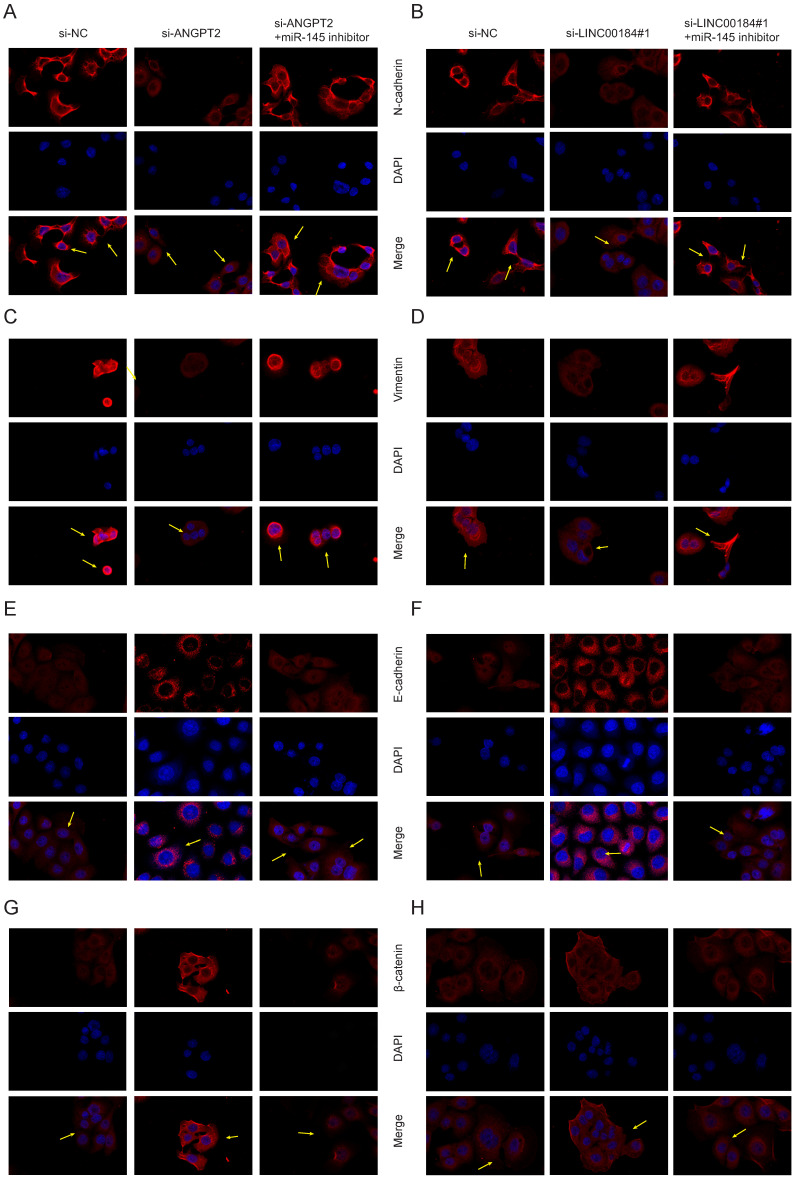
** LINC00184/miR-145/ANGPT2 axis regulate EMT in GC. A, C.** Levels of N-cadherin and Vimentin upon loss of ANGPT2 and inhibition of miR-145. **B, D.** Levels of N-cadherin and Vimentin upon loss of LINC00184 and inhibition of miR-145. **E, G.** E-cadherin and β-catenin upon reduction in ANGPT2 and blocking of miR-145. **F, H.** E-cadherin and β-catenin upon reduction in LINC00184 and blocking of miR-145.

As LINC00184/miR-145/ANGPT2 axis is correlated with the proliferation and invasion phenotypes of GC, we further analyzed its relationship with the regulation of EMT. The EMT markers were examined in si-ANGPT2, si-ANGPT2+miR-145 inhibitor, si-LINC00184 and si-LINC00184+miR-145 inhibitor conditions. Mesenchymal markers (N-cadherin and Vimentin) were inhibited following a decrease in ANGPT2, which was somewhat reversed by the inhibition of miR-145 (Fig. 7A, 7C). Though epithelial markers (E-cadherin and β-catenin) were increased by si-ANGPT2, and the inhibition of miR-145 rescued this phenomenon (Fig. 7E, 7G). Similarly, the knockdown of LINC00184 also inhibited mesenchymal markers and promoted epithelial markers. Finally, miR-145's inhibitors could partially reverse this (Fig. 7B, 7D, 7F, 7H). Arrows marked the changes of fluorescence intensity of membrane protein expression under different treatment conditions.

## Discussion

Through the advances in NGS technology and molecular biology, several thousand genes were shown to have a crucial function in the development of malignant tumors 16, 17 Also, it is gradually known that the importance of ncRNA in gene expression regulation 18, 19. Therefore, further understanding of the “regulatory network” between ncRNA and mRNA is very important to further understand the occurrence and development of GC 2, 20, 21. For our study, we acquired the transcriptome data of TCGA-STAD and set out to investigate the regulatory network of GC-related molecules through a series of bioinformatics analyses.

In order to find the target gene more accurately, we applied two methods of “limma” and “edgeR” to assess differentially expressed RNAs. Subsequently, we focused on overexpressed lncRNA, mRNA and weakly-expressed miRNA of GC in view of the negative regulatory association in ceRNA regulatory web. Finally, we constructed a ceRNA network containing 48 uDELs, 12 dDEMis and 29 uDEMs. GSEA and WGCNA were also analyzed. ANGPT attracted our attention because of its close association with cancer development.

ANGPT2, a ligand of receptor tyrosine kinase Tie-2, is mainly produced by endothelial cells which induces vascular instability and is a key factor of vascular maturation 22. In addition, ANGPT2 is often highly upregulated in the tumor vascular system compared to its low content in normal tissues 23. Elevated circulating ANGPT2 is closely associated with malignant invasion and worse prognosis in various cancers 24, 25. Besides, Jo MJ *et al.* 26 suggested that preoperative serum ANGPT2 levels correlated to lymph node metastasis in early GC patients. Other studies indicated that overexpression of ANGPT2 is associated with angiogenesis and malignant invasion in GC 27, 28. In the current study, we used a series of cell function assays to verify the carcinogenic function of ANGPT2 in enhancing the growth and invasive ability of GC cells and encouraging EMT. However, the regulatory mechanism of ANGPT2 still needs to be clarified in GC, particularly the function of ncRNAs that control ANGPT2. Since we discovered that miR-145 was upstream of ANGPT2, we then explored their biological roles in GC. MiR-145, located on chromosome 5q32, was weakly-expressed in multiple tumors and considered a tumor suppressor 29, 30. We demonstrated that miR-145 can particularly bind to the 3'UTR (Untranslated Region) of ANGPT2's mRNA, thereby blocking ANGPT2. Furthermore, the proliferation and invasion abilities of GC changed with miR-145 and ANGPT2 expression. The rescue experiment showed that the influence of miR-145 on GC malignancy was determined by changes in expression of ANGPT2. Compared to the inhibition of miR-145 on ANGPT2, our data shows a positive regulatory relationship among LINC00184 and ANGPT2, and the abnormal increase of LINC00184 could promote the proliferation and invasion of GC. Similar to ANGPT2, miR-145 can also inhibit the influence of LINC00184 on GC malignant phenotype. All these findings indicate the presence of a noteworthy regulatory network where miR-145 and LINC00184 cooperate and jointly control expression of ANGPT2.

Recently, the regulatory function of ncRNAs, particularly the lncRNAs, has attracted more and more attention. More and more evidences show that lncRNAs can competitively bind miRNA with mRNA to regulate the expression level of mRNA in cancers 12. For example, lncRNA-ATB acts as a ceRNA of miR-200 to control ZEB family in hepatocellular carcinoma 31. Exosomes lncRNA, LncARSR, stimulates resistance to sunitinib in renal cancer through the competitive binding of miR-34a and miR-449 32. Liu XH* et al.* 33 and Song YX *et al.* 4 respectively showed that the lncRNAs HOTAIR, KRTAP5-AS1 and TUBB2A lead to the occurrence and development of GC as ceRNAs. We found that LINC00184, as an oncogene, can be bound to miR-145 to influence ANGPT2 levels, thereby inducing the growth and invasion of GC.

**Figure 8:**
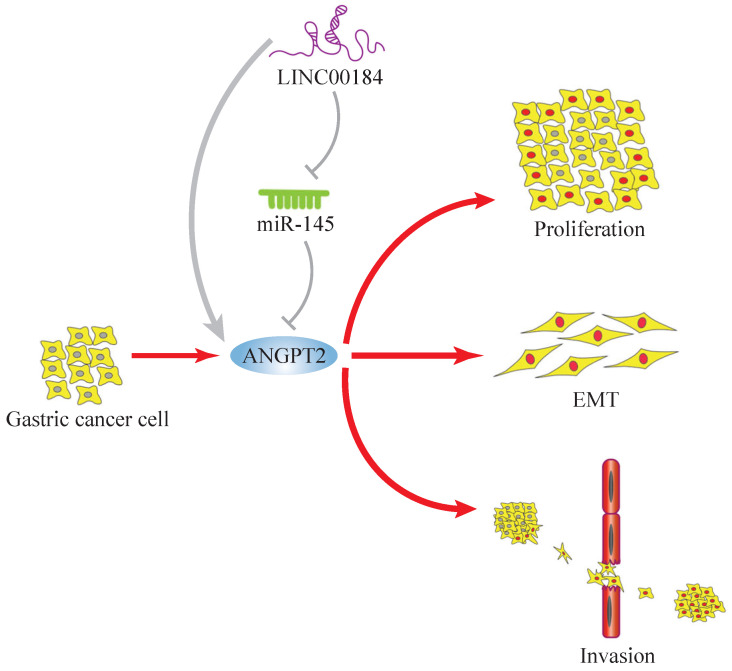
The mechanism graph of the regulatory network and function of LINC00184/miR-145/ANGPT2. ANGPT2 could promote proliferation, invasion and EMT processes of GC, which could be inhibited by miR-145 and enhanced by LINC00184 as ceRNAs.

In summary, genes do not usually act alone, so the importance of “networks” is self-evident. We introduced a LINC00184/miR-145/ANGPT2 regulatory network in GC. The molecular function of LINC00184 has been reported for the first time. ANGPT2 enhanced growth, invasive ability, and EMT of GC cells, while miR-145 partly rescued this process. Additionally, LINC00184 can increase the functions of ANGPT2 as a ceRNA (Fig. 8). These findings indicate that LINC00184/miR-145/ANGPT2 has a crucial function in the manifestation and progress of GC and can be a possible biomarker and target for GC therapy.

## Supplementary Material

Supplementary figures and tables.

## Figures and Tables

**Table 1:** The interaction between 48 uDELs and 13 dDEMis

dDEMis	uDELs
miR-100	DLX6-AS1
miR-137	MAL2, MUC19, TSSC1-IT1, ST7-AS2, LMO7-AS1, DLEU2, UFL1-AS1, POU6F2-AS2, LINC00299, HOTTIP, HULC, KCNQ1OT1
miR-143	C2orf48, PRSS30P, C15orf54, C10orf91, PSORS1C3, MUC19, UCA1, E2F3-IT1, TSSC1-IT1, HOTAIR, DLEU2, AC108463.1, LINC00460, LINC00494, CECR3, HOTTIP, PVT1, ARHGEF38-IT1, KCNQ1OT1, LINC00524.
miR-144	C8orf31 ,MUC19 ,DLEU2, DLX6-AS1, CASK-AS1 ,FNDC1-IT1
miR-145	MAL2, MUC19, LINC00184, LINC00337, ST7-AS2, LMO7-AS1, DLX6-AS1, UFL1-AS1, LINC00494, LINC00299, PVT1, PCAT1, KCNQ1OT1.
miR-187	MUC19, UBE2Q1-AS1, POU6F2-AS2, HOTTIP, PVT1, KCNQ1OT1
miR-195	C2orf48, RSS30P, C15orf54, MUC19, LINC00355, ABCA9-AS1, DLX6-AS1, NALCN-AS1, TSPEAR-AS1, DLEU7-AS1, HNF1A-AS1, CECR3, HOTTIP, PVT1, PCAT1, KCNQ1OT1.
miR-204	C2orf48, C8orf31, C10orf91, LINC00501, PSORS1C3, MUC19, ST7-AS2, LMO7-AS1, HOTAIR, UBE2Q1-AS1, ERVMER61-1, DLX6-AS1, LINC00299, HOTTIP, VCAN-AS1, HULC, ANO1-AS2, KCNQ1OT1, LINC00524.
miR-205	MUC19, LINC00184, ERVMER61-1, DLEU2, NALCN-AS1, AC108463.1, LINC00299, HOTTIP, PVT1, PCAT1, KCNQ1OT1, LINC00524.
miR-363	MAL2, C8orf31, LINC00501, C10orf126, MUC19, LINC00392, DLEU2, HNF1A-AS1, CECR3, PCAT1, KCNQ1OT1.
miR-383	C7orf69, MAL2, UCA1, LINC00337, DLX6-AS1, NALCN-AS1, POU6F2-AS2, LINC00494, LINC00299, PVT1, HULC, PCAT1.
miR-451	C10orf91, MUC19, NALCN-AS1
miR-497	C2orf48, PRSS30P, C15orf54, MUC19, LINC00355, ABCA9-AS1, DLX6-AS1, NALCN-AS1, TSPEAR-AS1, DLEU7-AS1, HNF1A-AS1, CECR3, HOTTIP, PVT1, PCAT1, KCNQ1OT1.

**Table 2:** The 9 dDEMis and the target 29 uDEMs

dDEMis	uDEMs
miR-100	HS3ST2
miR-137	SLC1A5, CSE1L
miR-143	COL5A2, COL1A1, SERPINE1
miR-144	HOXA10, KPNA2
miR-145	ANGPT2, SERPINE1, MEST
miR-195	MYB, CLSPN, OSBPL3, ITGA2, ATP13A3, KIF23, CBX2, CCNE1, CEP55, CHEK1, HOXA10, E2F7, OSCARCDC25A
miR-204	IL11, HOXC8
miR-363	MYO1B, SOX11, SOX4, GOLGA8A
miR-497	CHEK1, CEP55, HOXA10, OSBPL3, CLSPN, OSCAR, ITGA2, KIF23, E2F7, CBX2, CCNE1, CDC25A
