# Supplementary material for: LINC00184 involved in the regulatory network of ANGPT2 via ceRNA mediated miR-145 inhibition in gastric cancer
Source: J Cancer. 2021 Feb 22;12(8):2336–50. doi: 10.7150/jca.49138 (PMC7974899; doi:10.7150/jca.49138)
Supplement: Supplementary file 1 — Supplementary figures and tables. [file jcav12p2336s1.pdf]

Supplement Table 1. The sequence information of the biomarkers

	Orientation	Sequence (5' to 3')
ANGPT2	Forward	GTGATTGATTCGGATACTGAC
	Reverse	CTGGACCTGATATTGCTTCT
miR-145	reverse	GTCGTATCCAGTGCAGGGTCCGAGGTATT
	transcription	CGCACTGGATACGACAGGGAT
	Forward	GCGGCGGGTCCAGTTTTCCCAG
	Reverse	ATCCAGTGCAGGGTCCGAGG
LINC00184	Forward	TGAGAAGCAAGGAAGAGAC
	Reverse	GTAGATGACGGCACAGTT
U6	Forward	GCTTCGGCAGCACATATACTAA
	Reverse	AACGCTTCACGAATTTGCGT
si-ANGPT2-1	SS Sequence	GAAAGACAACAACAAGUUUCU
	AS Sequence	AAACUUGUUGUUGUCUUUCGA
si-ANGPT2-2	SS Sequence	GGAUGGAGACAACGACAAAUG
	AS Sequence	AAACUUGCACAUAACAUUCUU
si-LINC00184-1	SS Sequence	GCUUAAUGCAGUUAAGUAACA
	AS Sequence	UUACUUAACUGCAUUAAGCUG
si-LINC00184-2	SS Sequence	GACAAGUGUUGAAGGUGAAAG
	AS Sequence	UUCACCUUCAACACUUGUCUU

Supplement Figure 1 *Differentially expressed RNAs in GC*

A-C Heatmaps of top 50 DELs, DEMis and DEMs, identified by edgeR.

D-F. Volcano plots of the expression levels of differentially expressed DELs, DEMis and DEMs, identified by edgeR.

G-I. PCA of DELs, DEMis and DEMs, by edgeR.

J. The down-regulated DELs determined by limma and edgeR.

K. The up-regulated DEMis determined by limma and edgeR.

L. The down-regulated DEMs determined by limma and edgeR.

Supplement Figure 2 *GO analysis*

A-E: The top 5 biological processes (BP).

F-J: The top 5 molecular functions (MF).

K-O: The top 5 cellular components (CC).

Supplement Figure 3 *The top 10 KEGG pathways.*

A. cell cycle; B. DNA replication; C. homologous recombination; D. mismatch repair; E. nucleotide excision repair; F. base excision repair; G. RNA degradation; H. one carbon pool by folate; I. ECM receptor interaction; J. spliceosome.**Supplement Figure 4**

A. The soft-thresholding power used to construct scale-free network.

B. Visualization of hierarchical clustering dendrogram of the eigengenes (up). Heatmap of the eigengene adjacency (down).

Supplement Figure 5

A, B. RT-PCR showed ANGPT2 knockdown in comparison to scrambled control in GC cells.

C, D. PCGEM1 in the cellular portions of SGC-7901s and BGC-823s.

E, F. RT-PCR showed the efficiency of LINC00184 knockdown in comparison to scrambled control in GC cells.

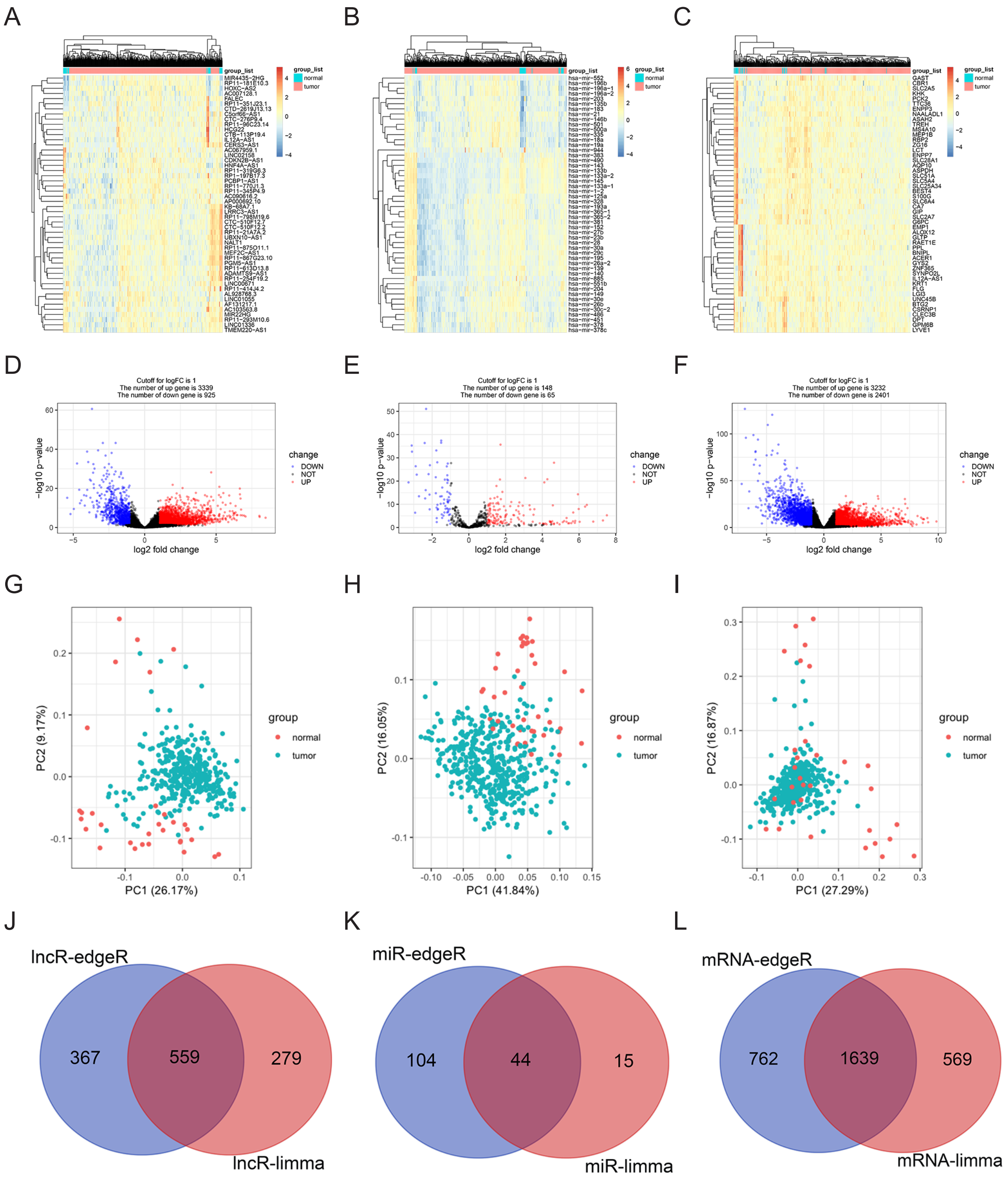
A
B
C
D
E
F
G
H
I
J
K
L
Cutoff for logFC is 1
The number of up gene is 3339
The number of down gene is 925
Cutoff for logFC is 1
The number of up gene is 148
The number of down gene is 65
Cutoff for logFC is 1
The number of up gene is 3232
The number of down gene is 2401
change
DOWN
NOT
UP
−log10 p−value
log2 fold change
group
normal
tumor
PC1 (26.17%)
PC2 (9.17%)
PC1 (41.84%)
PC2 (16.05%)
PC1 (27.29%)
PC2 (16.87%)
lncR-edgeR
lncR-limma
367
559
279
miR-edgeR
miR-limma
104
44
15
mRNA-edgeR
mRNA-limma
762
1639
569

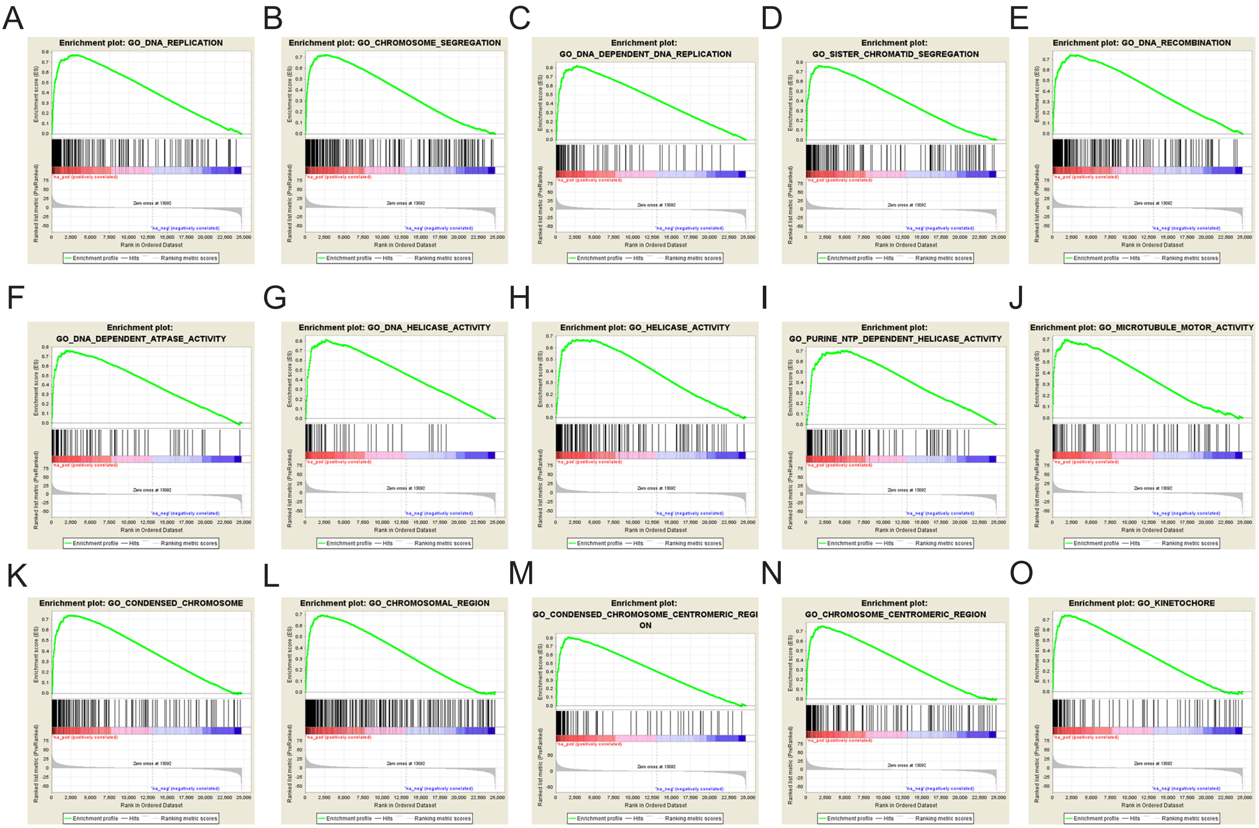
A
Enrichment plot: GO_DNA_REPLICATION
B
Enrichment plot: GO_CHROMOSOME_SEGREGATION
C
Enrichment plot: GO_DNA_DEPENDENT_DNA_REPLICATION
D
Enrichment plot: GO_SISTER_CHROMATID_SEGREGATION
E
Enrichment plot: GO_DNA_RECOMBINATION
F
Enrichment plot: GO_DNA_DEPENDENT_ATPASE_ACTIVITY
G
Enrichment plot: GO_DNA_HELICASE_ACTIVITY
H
Enrichment plot: GO_HELICASE_ACTIVITY
I
Enrichment plot: GO_PURINE_NTP_DEPENDENT_HELICASE_ACTIVITY
J
Enrichment plot: GO_MICROTUBULE_MOTOR_ACTIVITY
K
Enrichment plot: GO_CONDENSED_CHROMOSOME
L
Enrichment plot: GO_CHROMOSOMAL_REGION
M
Enrichment plot: GO_CONDENSED_CHROMOSOME_CENTROMERIC_REGION
N
Enrichment plot: GO_CHROMOSOME_CENTROMERIC_REGION
O
Enrichment plot: GO_KINETOCHORE
Enrichment score (ES)
Ranked list metric (PreRanked)
Rank in Ordered Dataset
'na_pos' (positively correlated)
'na_neg' (negatively correlated)
Zero cross at 13092
Enrichment profile
Hits
Ranking metric scores

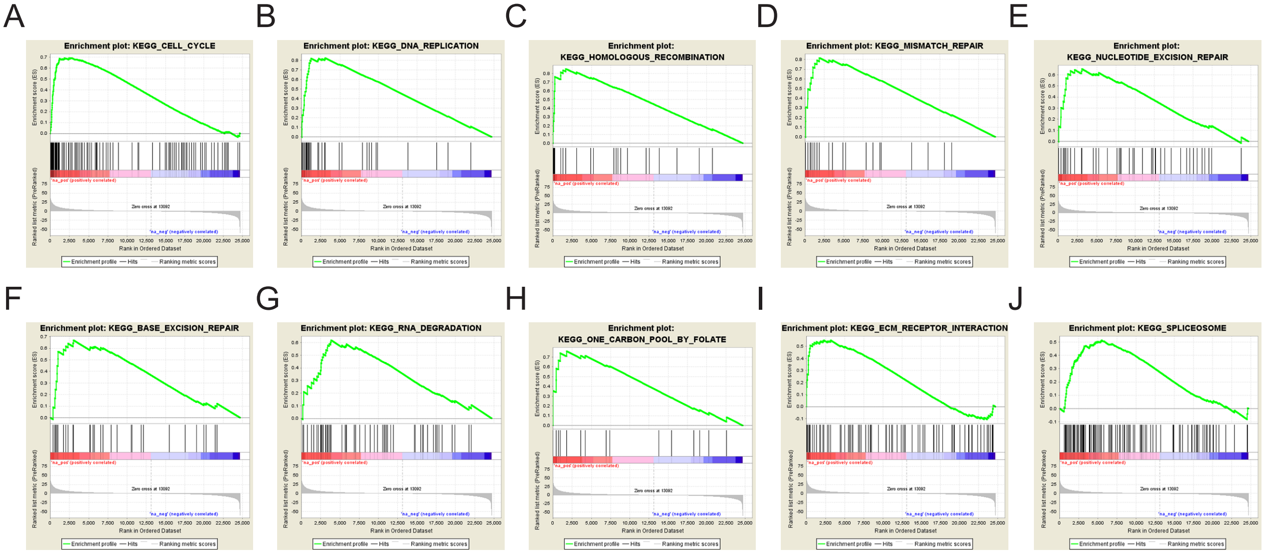

A
Enrichment plot: KEGG_CELL_CYCLE
B
Enrichment plot: KEGG_DNA_REPLICATION
C
Enrichment plot: KEGG_HOMOLOGOUS_RECOMBINATION
D
Enrichment plot: KEGG_MISMATCH_REPAIR
E
Enrichment plot: KEGG_NUCLEOTIDE_EXCISION_REPAIR
F
Enrichment plot: KEGG_BASE_EXCISION_REPAIR
G
Enrichment plot: KEGG_RNA_DEGRADATION
H
Enrichment plot: KEGG_ONE_CARBON_POOL_BY_FOLATE
I
Enrichment plot: KEGG_ECM_RECEPTOR_INTERACTION
J
Enrichment plot: KEGG_SPLICEOSOME
Enrichment score (ES)
Ranked list metric (PreRanked)
'na_pos' (positively correlated)
Zero cross at 13092
'na_neg' (negatively correlated)
Rank in Ordered Dataset
Enrichment profile
Hits
Ranking metric scores

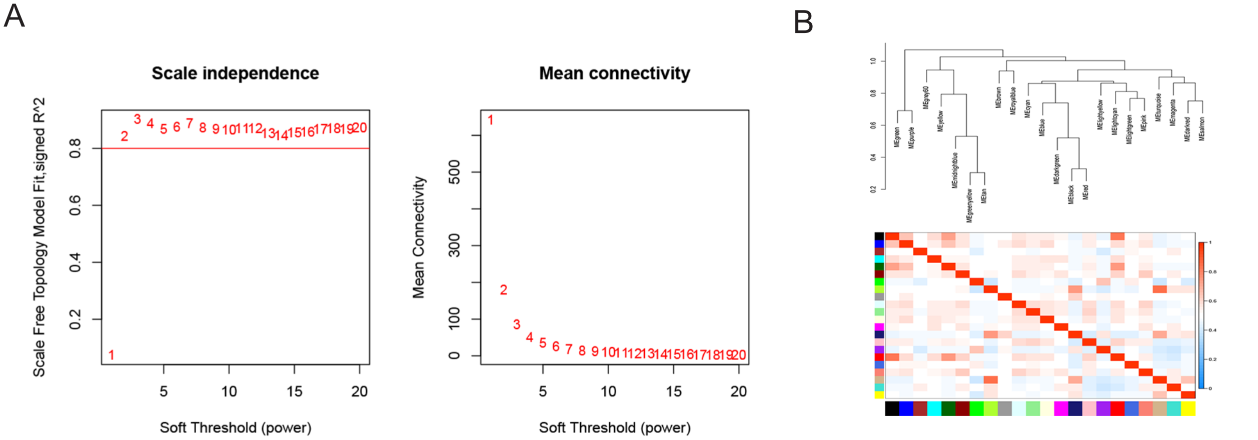
A
Scale independence
Scale Free Topology Model Fit,signed R^2
Soft Threshold (power)
Mean connectivity
Mean Connectivity
Soft Threshold (power)
B
MEgreen
MEpurple
MEgrey60
MEyellow
MEmidnightblue
MEgreenyellow
MEtan
MEbrown
MEroyalblue
MEcyan
MEblue
MEdarkgreen
MEblack
MEred
MElightyellow
MElightcyan
MElightgreen
MEpink
MEturquoise
MEmagenta
MEdarkred
MEsalmon

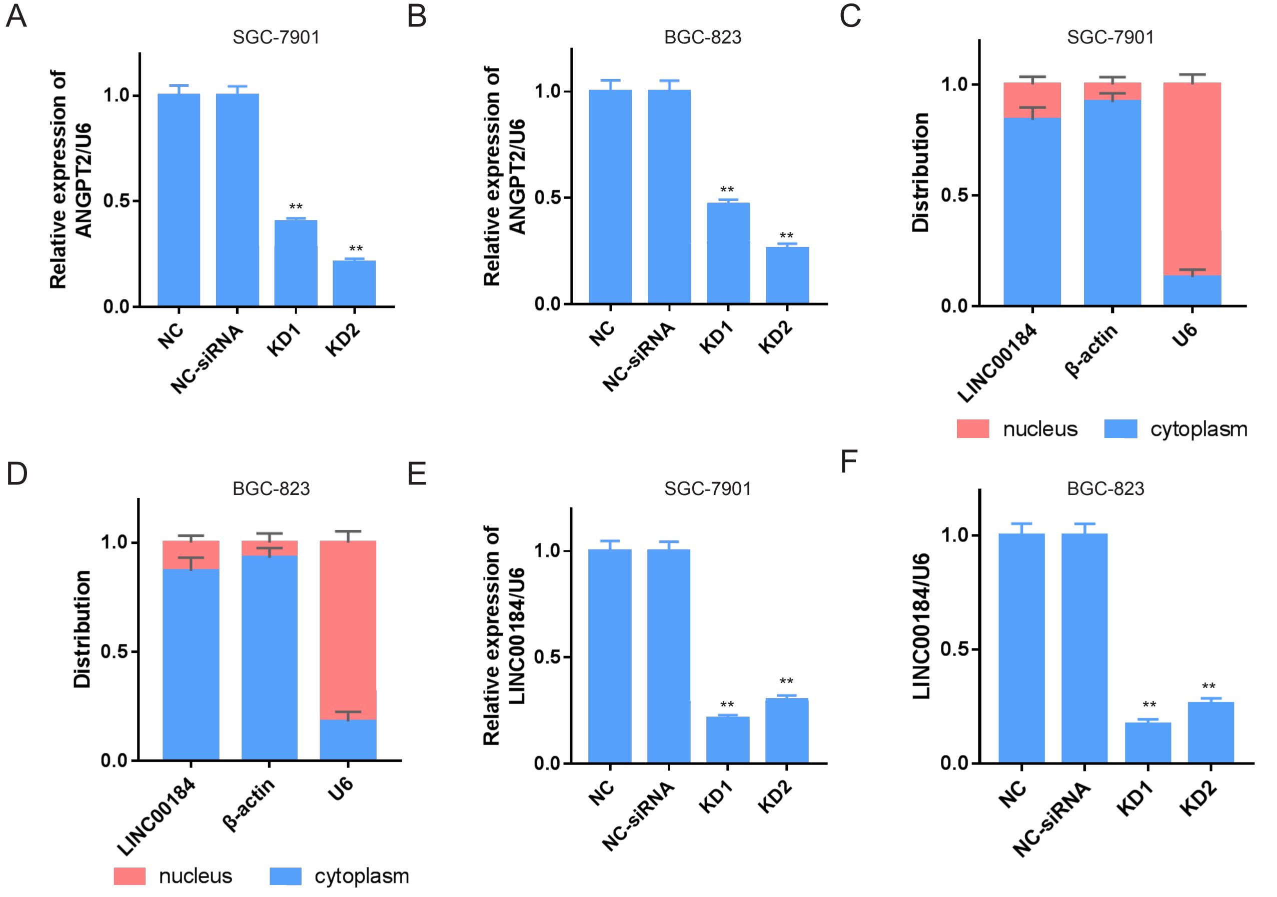
A
SGC-7901
Relative expression of ANGPT2/U6
1.0
0.5
0.0
**
**
NC
NC-siRNA
KD1
KD2
B
BGC-823
Relative expression of ANGPT2/U6
C
SGC-7901
Distribution
LINC00184
β-actin
U6
nucleus
cytoplasm
D
BGC-823
Distribution
E
SGC-7901
Relative expression of LINC00184/U6
F
BGC-823
LINC00184/U6
